# *In situ* examination of *Lactobacillus brevis* after exposure to an oxidizing disinfectant

**DOI:** 10.3389/fmicb.2014.00623

**Published:** 2014-11-26

**Authors:** Yu Zhao, Susanne Knøchel, Henrik Siegumfeldt

**Affiliations:** Food Microbiology, Department of Food Science, Faculty of Science, University of CopenhagenFrederiksberg C, Denmark

**Keywords:** *Lactobacillus brevis*, aerobic cultivation, anaerobic cultivation, peracetic acid, solid surface, microscopic method, heterogeneity

## Abstract

Beer is a hostile environment for most microorganisms, but some lactic acid bacteria can grow in this environment. This is primarily because these organisms have developed the ability to grow in the presence of hops. It has been speculated that hop resistance is inversely correlated to resistance against oxidation, and this would have great impact on the use of various disinfectants in the brewing industry. In this study, we cultivated bacteria under aerobic and anaerobic conditions, and then investigated the *in situ* outgrowth of individual cells into microcolonies on de Man Rogosa Sharpe (MRS) agar after exposure to the oxidizing agent peracetic acid (PAA). An automated microscope stage allowed us to analyse a much larger number of cells over extended periods of incubation. After PAA treatment, the lag time increased markedly, and extensive variation in morphology, μ_max_ as well as stress resistance was observed between and within the tested *Lactobacillus brevis* strains. The results suggest that aerobic cultivation increased the oxidative stress tolerance in *Lactobacillus brevis*. The results also show that dead cells are randomly distributed in a microcolony and the majority of non-growing individual cells do not stain with a membrane impermanent dye (Propidium iodide), which indicates that PAA may not destroy the plasma membrane. In conclusion, the developed microscopic analysis of individual cells on MRS agar can provides faster results and more details of cell physiology compared to the traditional CFU method.

## INTRODUCTION

Lactic acid bacteria (LAB) are fermentative organisms which have been generally regarded as anaerobic bacteria, but most of them can grow under aerobic conditions (Sakamoto and Komagata, 1996). Beer is a relatively hostile medium for most microorganisms. The low pH will prevent most Gram-negative bacteria from growing, and the addition of hops will usually prevent LAB from spoiling the beer (Vaughan et al., 2005; Suzuki et al., 2006; Menz et al., 2010; Suzuki, 2011). However, some LAB possess a level of hop resistance, and therefore also possess the ability to spoil beer. 60–90% of bacteria isolated from spoiled beer are LAB (Suzuki, 2011). Among those LAB, *Lactobacillus brevis* is the most common bacteria and frequently detected in breweries (Hollerová and Kubizniaková, 2001; Suzuki et al., 2008a; Menz et al., 2010).

Recently, it has been suggested that hop resistance in LAB is inversely correlated to resistance toward oxidative compounds (Behr and Vogel, 2010). Consequently, it would be interesting to investigate the response of beer spoilage isolates toward oxidative compounds, as some of these are utilized as sanitizers in the food industry (Rossoni and Gaylarde, 2000; Kitis, 2004).

Another potential challenge is the relatively slow growth of the beer spoilage organisms. This means that often they are not detected readily in various culture media (Suzuki et al., 2008b). One way of facilitating the detection would be to use detection of growth of individual cells into micro-colonies, as the formations of macrocolonies require a longer incubation period in traditional CFU method. Recently, some studies have developed bioimaging methods for detecting the growth of individual cells in/on a solid matrix. Elfwing et al. (2004) designed a flow chamber microscopic method to observe growth and proliferation of single cells of *Escherichia coli* and *Listeria innocua*. Niven et al. (2006) developed a phase-contrast microscopy method to determine the first division time and individual lag times on agar media. Mertens et al. (2012) studied the colony growth dynamics based on optical density measurements on solid medium in microtiter plates. Koutsoumanis and Lianou (2013) used time-lapse microscopy videos to count the cells and to observe the division of *Salmonella* single cells directly on agar media. Ryssel et al. (2013) developed a microscopy method to monitor growth and death of individual *Lactococcus lactis* cells based on staining with propidium iodide (PI) in the agar media. In addition, another advantage of investigating individual cells growing on a solid substrate is the ability to analyze the heterogeneity of a given population, as each individual cell gives rise to a unique microcolony.

The current study therefore investigates the impact of oxidizing substances on the survival of beer spoilage LAB. The study describes an automated image-acquisition microscopic method that enables the analysis of growth as well as the death of individual cells while growing on the surface of a semisolid substrate.

## MATERIALS AND METHODS

### BACTERIAL STRAINS AND GROWTH CONDITIONS

The strains of *Lactobacillus brevis* used in this study are listed in **Table 1**. All experiments were initiated by inoculating 10 ml de Man Rogosa Sharpe (MRS) broth (Merck, pH 5.7) from a frozen stock culture, followed by incubation at 30°C overnight. Subsequently, 100 μl culture was subcultured into 10 ml fresh MRS broth. For aerobic cultivation, the tubes was shaken around 300 rpm at 30°C. For anaerobic cultivation, the tubes were incubated at 30°C in an anaerobic jar, and incubated until an approximate OD_600_ value of 1.5. The cultures were subsequently exposed to oxidizing agents as described below.

**Table 1:** Overview of the strains used in this study.

Abbreviation	Strain	origin	Type
JK09	*Lactobacillus brevis* JK09	Danish craft beer	Wild type
JK09 - horA	*Lactobacillus brevis* JK09 - horA*	Danish craft beer	Plasmid cured
MI2158	*Lactobacillus brevis* MI2158	DSM20054T	Wild type
HF01	*Lactobacillus brevis* HF01	Danish craft beer	Wild type
HF02	*Lactobacillus brevis* HF02	Danish craft beer	Wild type

*This strain was cured from the horA plasmid.

### TREATMENTS OF LAB WITH OXIDIZING AGENTS

Two kinds of disinfectants were used in this study: peracetic acid (PAA, Sigma, 101272695) and sodium hypochlorite (NaClO, Sigma, 101292621). The final concentration of PAA solution during exposure was 0.0014%, and the final concentration of NaClO solution was 0.0021%. 0.5 ml of the aerobic cultures or anaerobic cultures were added into three 15 mm × 18 cm glass tubes containing either 12 ml saline (control), 12 ml PAA solution or 12 ml NaClO solution, mixed with a whirlimixer for 20 s and leave them for 10 min at 23°C. Subsequently, 0.5 ml of each cell suspension was diluted into 4.5 ml saline and mixed as described above in order to rapidly reduce the toxicity of oxidizing agents greatly (Grönholm et al., 1999). Subsequently, the surviving cells were enumerated by CFU.

### DETERMINATION OF CFU

Cell suspensions were serially diluted in saline (0.9%, pH 5.8) and transferred to MRS agar plates (Merck), then incubated at 30°C for 5 days, with analysis on day 3–5.

### MICROSCOPIC METHOD

Peracetic acid was chosen as the oxidizing agent for microscopic analysis, but in order to reduce the number of killed cells, the concentration of PAA was decreased to 0.001%. The treatment was otherwise the same as previously described.

The microscope set-up was the same as described by Ryssel et al. (2013). After the treatment with PAA, 5 μl of the cell suspension was transferred to the bottom of a well in an Ibidi μ-Slide 8-well chamber (hydrophobic, uncoated, sterile, ibidi GmbH, München, Germany). The dead cell impermanent dye PI (Molecular Probes, Invitrogen, Oregon) was previously added to molten MRS agar to a final concentration of 2.0 μg/ml at 45°C, and 300 μl of the molten MRS-PI agar medium was added slowly to the well (to prevent the cells from leaving the bottom surface). The addition of MRS-PI agar constituted time zero for the experiment. After the agar solidified, the chamber was placed in the automated microscope stage, and a random spot was chosen in each well as the starting position. Subsequently, a total of 49 positions were recorded in a 7 × 7 grid, which was programmed into the software. The pre-programmed grid was used to avoid user bias when selecting appropriate spots in the specimen. Furthermore, the 49 positions enabled us to obtain more valid information about the heterogeneity of the specimen. The recording of all 49 positions were then repeated throughout the experiment. Brightfield images and PI fluorescent images were captured using MetaMorph 7.0 software package (Molecular Devices Inc., Silicon Valley, CA, USA). The chamber was left on the microscope stage at 23°C during the entire experiment.

### DATA ANALYSIS

#### Calculation of log reduction

Log reduction was defined as the difference between the log CFU count of saline treatment (control) and disinfectant treatment.

#### Calculation of survival in percentage

In order to compare the microscopic analysis and the CFU, the survival percentage was calculated as follows.

$$S\,u\,r\,v\,i\,v\,a\,l_{C\,F\,U}=C\,F\,U_{T\,r\,e\,a\,t\,m\,e\,n\,t}/C\,F\,U_{C\,o\,n\,t\,r\,o\,l}\,\left(T\,r\,e\,a\,t\,m\,e\,n\,t\,\;\;i\,s\,\;\;c\,o\,n\,t\,r\,o\,l\,\;\;t\,r\,e\,a\,t\,m\,e\,n\,t\,\;\;o\,r\,\;\;P\,A\,A\,\;\;\;t\,r\,e\,a\,t\,m\,e\,n\,t\right)$$

$$S\,u\,r\,v\,i\,v\,a\,l_{m\,i\,c\,r\,o\,s\,c\,o\,p\,e}=N\,u\,m_{d\,i\,v\,i\,d\,i\,n\,g\,\;\;c\,e\,l\,l\,s}/N\,u\,m_{T\,o\,t\,a\,l\,\;\;c\,e\,l\,l\,s\,\;\;a\,t\,\;\;t\,i\,m\,e\,\;\;z\,e\,r\,o}$$

#### Cell size analysis

The brightfield images were analyzed with the free image analysis software image J [version 1.48; National Institutes of Health (NIH), Bethesda, MD, USA^1^]. Before cell division occurs, the size of individual cells is measured directly by pixels, but after cell division, the areas of the growing microcolonies were measured.

#### Calculation of lag time and μ_max_

Growth data (time and cell size) were analyzed using the DMFit software available on the Combase website^2^. Growth data were fitted to the model proposed by Baranyi and Roberts (1994) for estimation of lag time (λ, hour) and maximum specific growth rates (μ_max_, Ln pixels/hour) of each growth curve.

## RESULTS

### EFFECT OF OXIDIZING AGENTS ON LAB

**FIGURE 1:**
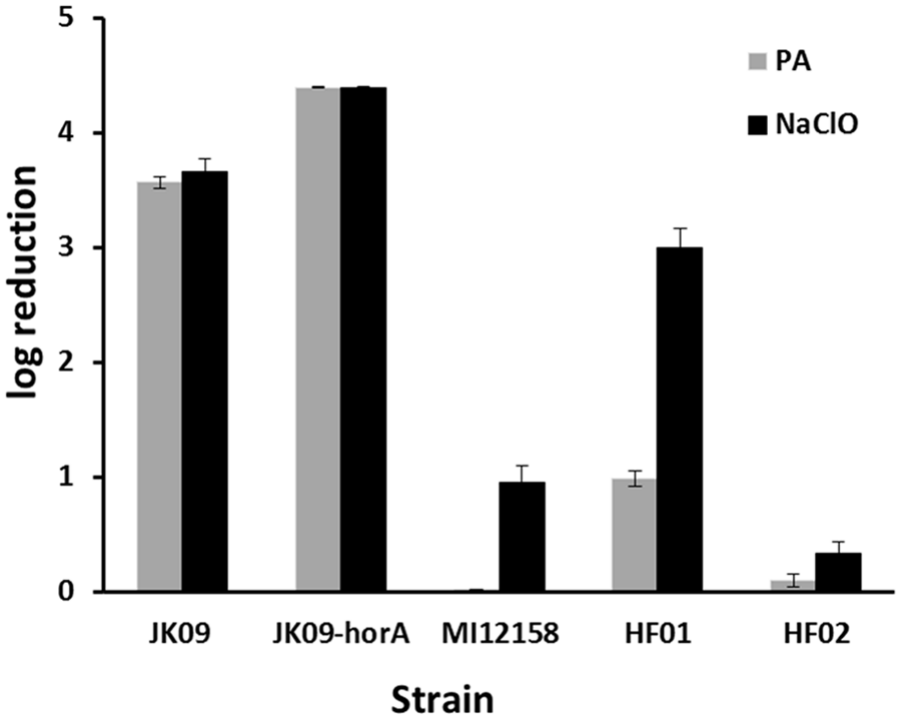
**Log reduction of five *Lactobacillus brevis* strains after exposure to 0.0014% peracetic acid (PAA; gray bars) and 0.0021% NaClO (black bars).** The smaller the log reduction, the bigger the tolerance.The error bars indicate the SD.

**Figure 1** shows the log reduction after treatment with PAA and NaClO on five *Lactobacillus brevis* strains. The initial log CFU of all the strains after anaerobic cultivation was 8.72 ± 0.10 (mean value ± SD). The two disinfectants exhibited different effectiveness against the different strains. After PAA treatment, JK09-hor A, which is a plasmid-cured strain, was the most sensitive strain, while the non-beer associated bacteria MI12158 was the most tolerant one; for NaClO treatment, JK09-hor A was still the most sensitive and HF02 was the most tolerant.

The two strains HF01 and JK09 were subsequently chosen for further experiments in a microscopic set-up, because they are both beer spoilage bacteria, and exhibited varying levels of sensitivity toward PAA, which was selected as the oxidizing agent for the microscopic analysis. The isolate HF02 was very tolerant toward the oxidizing agents, which would impede the microscopic analysis, and on the other hand, NaClO did not produce a pronounced difference between HF01 and JK09.

### GROWTH UNDER AEROBIC AND ANAEROBIC CULTIVATIONS

Both HF01 and JK09 grew better under aerobic cultivation. For HF01, the aerobic culture reached OD_600_ of 1.5 after16h and the anaerobic culture after 21h. For JK09, the time was 14h and 18h under aerobic and anaerobic cultivations, respectively. In addition, the pH at OD_600_ of 1.5 under aerobic and anaerobic cultivations were 4.91 and 4.94 for HF01, whereas the pH of JK09 were 4.85 and 4.93, respectively.

### COMPARISON BETWEEN CFU AND MICROCOLONY FORMATION

**FIGURE 2:**
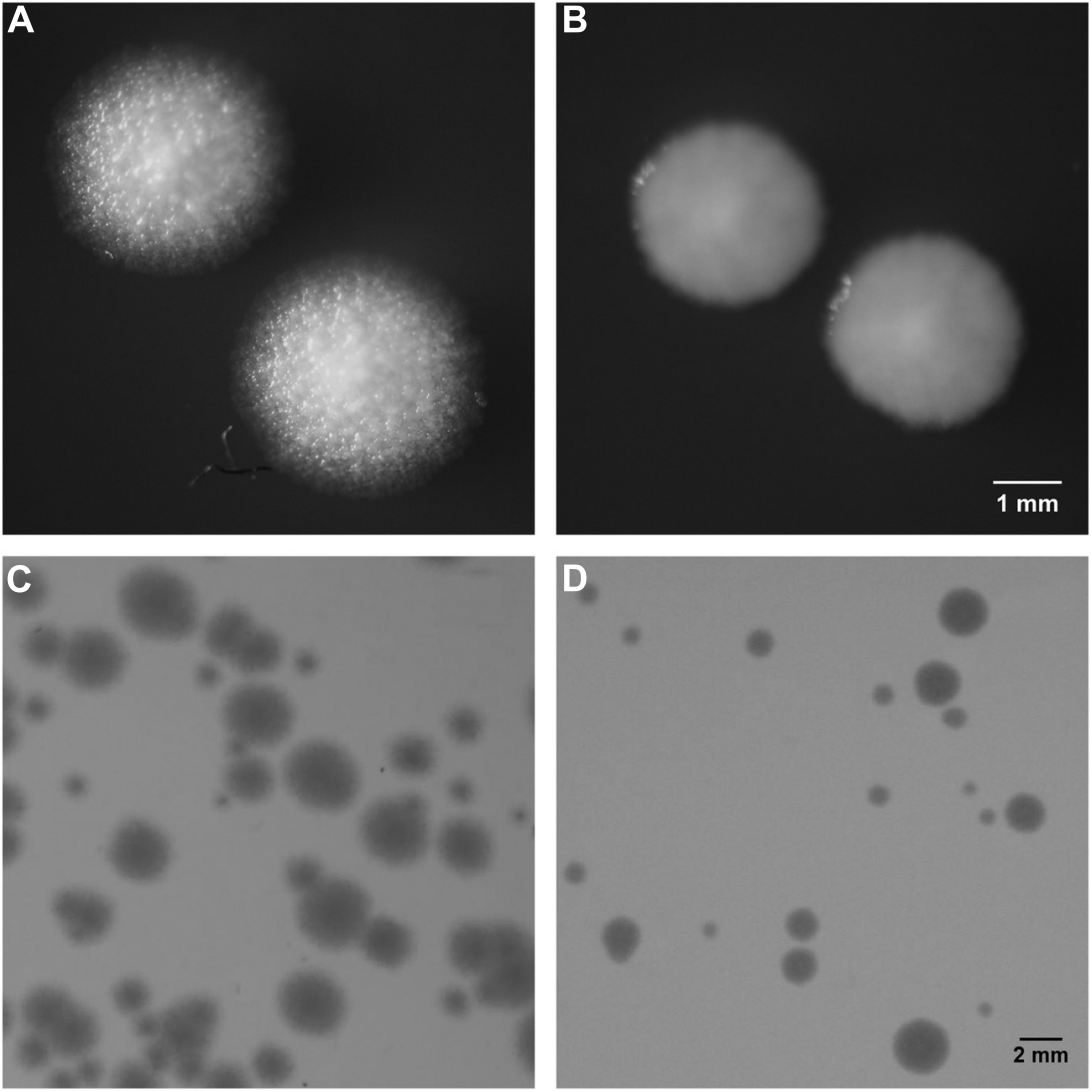
**Colony morphology of HF01 and JK09 on MRS agar after anaerobic cultivation.** The colonies were photographed after 5 days of incubation. HF01 control **(A)**, JK09 control **(B)**, HF01 exposed to PAA **(C)**, JK09 exposed to PAA **(D)**. Strain HF01 grows as rough colonies while JK09 grows as smooth colonies. For both HF01 and JK09, the colonies without treatment were uniform in size, in contrast, the colonies after treatment with PAA were varying in size. **(A)** and **(B)** were illuminated from above to highlight the surface structure; **(C)** and **(D)** were illuminated from below to visualize the size difference of the colonies.

For all treatments, colony forming units were detected up to 5 days by traditional CFU method, and microcolonies were observed for up to 2 days in the microscopic method. In the control experiments of HF01 and JK09, the size of the individual (macro)colonies on the plates was comparatively large and very similar and the number of colonies would not increase after 3 days of incubation. In contrast, the number of colonies increased for up to 5 days after exposure to PAA, and the size of colonies were heterogenous, since some colonies were as large as in the control, and other colonies were still much smaller on day 5. **Figure 2** is an example of colony morphologies after anaerobic cultivation, the colony morphologies were similar after aerobic cultivation (results not shown).

**FIGURE 3:**
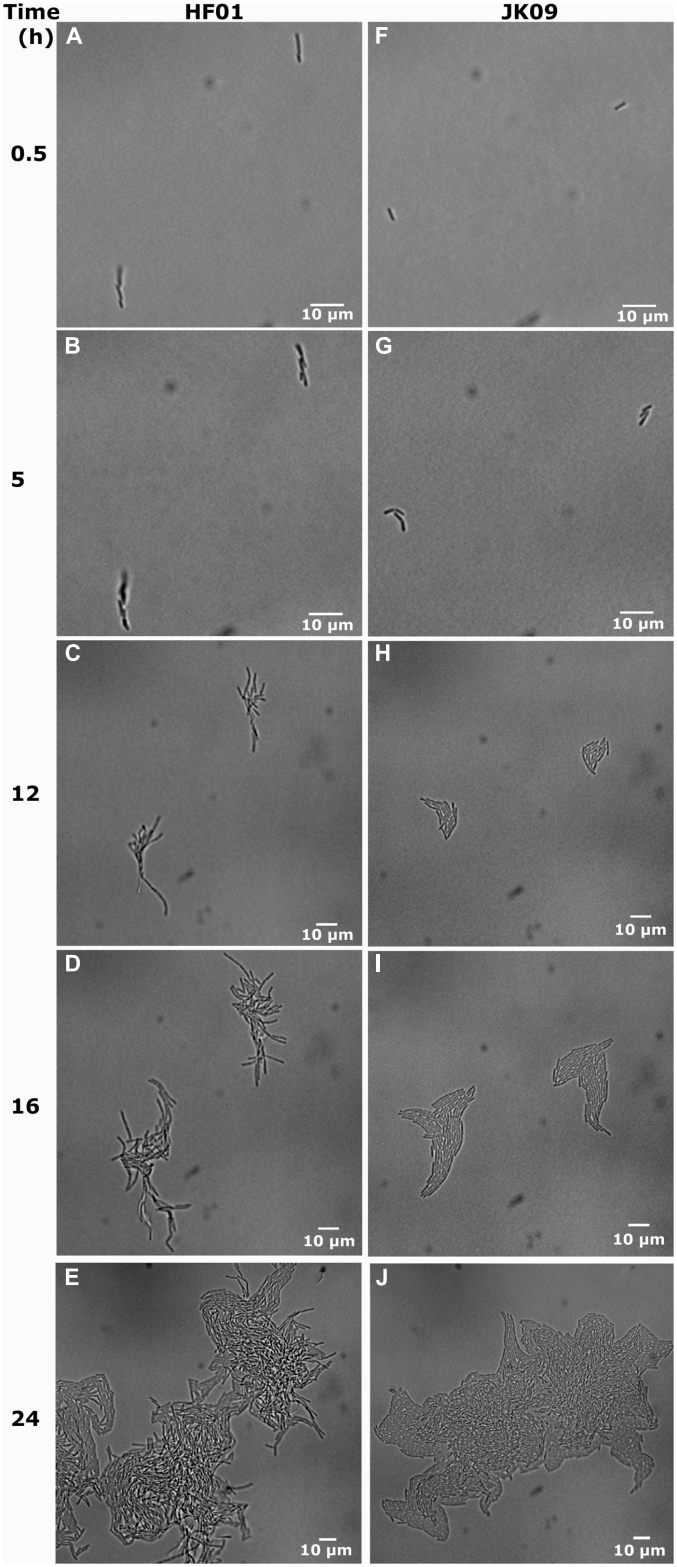
**Development of individual cells for HF01 control and JK09 control on MRS agar after anaerobic cultivation. (A)**, **(B)**, **(F)**, **(G)** have the same magnification; **(C)**, **(D)**, **(H)**, **(I)** have the same magnification and **(E)**, **(J)** have the same magnification.

A clear difference in colony morphology between HF01 and JK09 could be observed both on the plates (**Figures 2A,B**) and in the microscope (**Figure 3**). For HF01, the surface of (macro)colonies was rough and the edge appeared fluffy (**Figure 2A**). In the microscope, the microcolonies did not develop in all directions equally, but in a more random fashion and there were sometimes empty space within a microcolony (**Figure 3E**). For JK09, the surface of the (macro)colonies was smoother, and the edge was rounder (**Figure 2B**), with the growth of the microcolony expanding more equally in all directions (**Figure 3H**).

Although we conventionally assume that all untreated cells (i.e., control) would grow and form colonies, it was observed that some individual cells never started to divide under the microscope (**Figure 6**), and therefore the calculation of the survival in the microscopic method will not reach 100%.

**Table 2** shows that there is good reproducibility of both methods, although the variation between repetitions of CFU could be up to 17.4%, where the variation between repetitions of the microscopic method was up to 12.8%. In general, for the control, the survival of the CFU method is slightly higher than that of the microscopic method, from 1.9% to 7.1%. But in most cases, after PAA, the survival of the microscopic method is higher, from -0.7% to 25.9%. We can also see from **Table 2** that after exposure to PAA, the survival rate of HF01 was always higher than that of JK09 regardless of cultivation, and the survival rates of both HF01 and JK09 after aerobic cultivation were significantly higher than after anaerobic cultivation.

**Table 2:** Comparison of survival rate measured by CFU and microscopic method.

Strain	Cultivation	repetition	Treatment	CFU method %	Microscopic method %
HF01	Anaerobic	1	Control	100.0	93.6
			PAA	39.7	38.6
		2*	Control	100.0	92.9
			PAA	47.4	51.4
	Aerobic	1	Control	100.0	97.8
			PAA	72.4	94.2
		2*	Control	100.0	97.1
			PAA	85.7	96.5
JK09	Anaerobic	1	Control	100.0	97.5
			PAA	1.9	1.3
		2*	Control	100.0	98.1
			PAA	1.0	0.3
	Aerobic	1	Control	100.0	97.8
			PAA	32.1	58.0
		2*	Control	100.0	98.6
			PAA	49.5	55.6

*Experiments which were used to draw the growth curves of individual cells in **Figure 5**.

### MICROSCOPIC METHOD

In **Figure 3**, we show the universal behavior of individual cells in the control experiments after anaerobic cultivation. Usually the cells elongate to two or three times the initial length, where after we observe the division into two or three cells. The cells continue to multiply, and eventually form a microcolony. We also observed a few cells that increased in cell length up to six times the initial length before division, while very few other cells prolonged a little but never started dividing (**Figure 6**).

**FIGURE 4:**
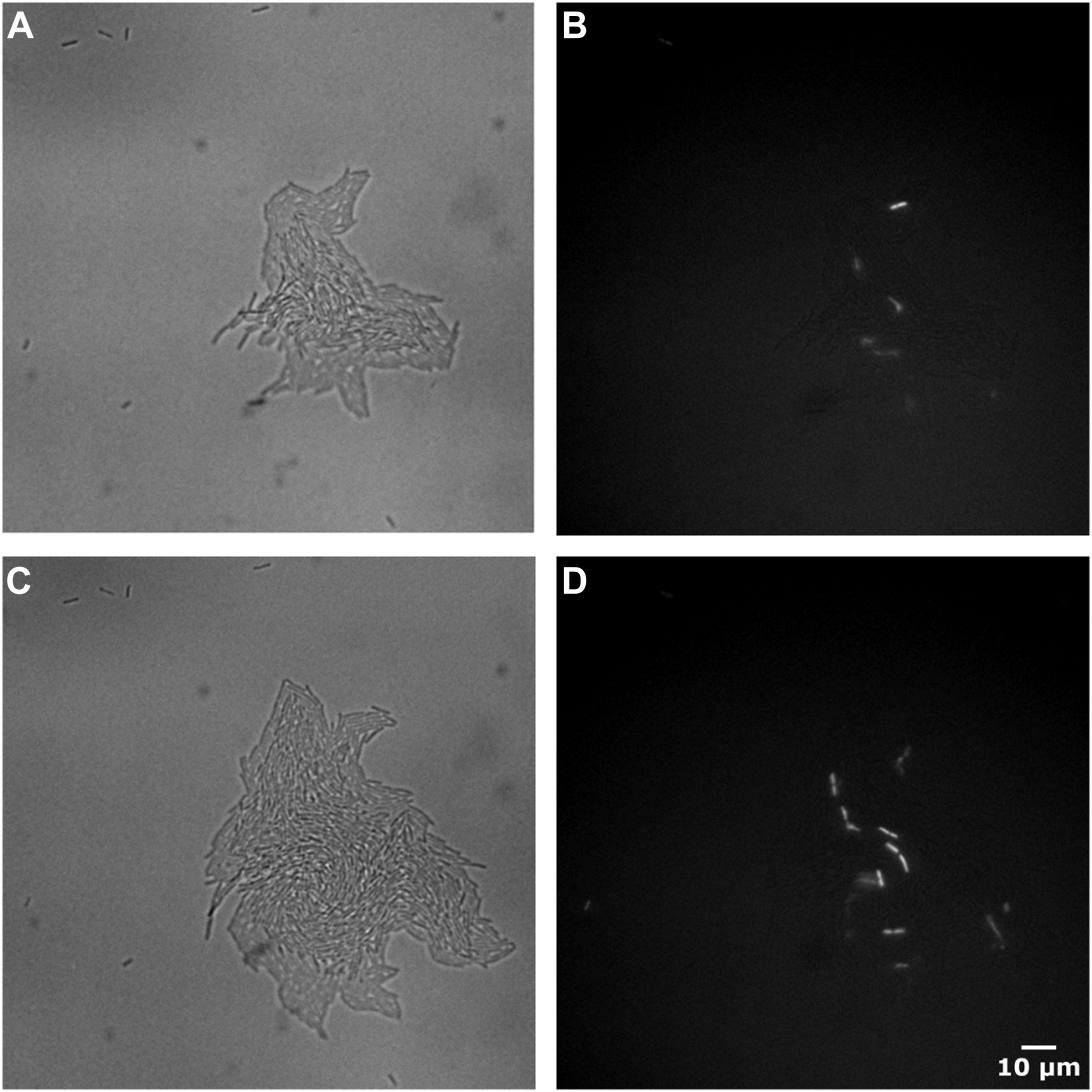
**Images of brightfield and PI fluorescent of JK09 after anaerobic cultivation followed by exposure to PAA.** Brightfield at 44 h **(A)**, propidium iodide (PI) fluorescent at 44 h **(B)**, brightfield at 48 h **(C)**, PI fluorescent at 48 h **(D)**.

Staining with PI did not by itself affect viability of *Lactobacillus brevis* (results not shown). Examples of corresponding brightfield and PI images of JK09 after anaerobic cultivation followed by PAA treatment are shown in **Figure 4**. At 44 h, there are some red cells randomly distributed inside the microcolony (**Figure 4B**). But only two cells out of the initial seven non-growing cells were red. After 4 h, the number of red cells increased within the extending microcolonies, and one additional individual cell turned red (**Figure 4D**).

**FIGURE 5:**
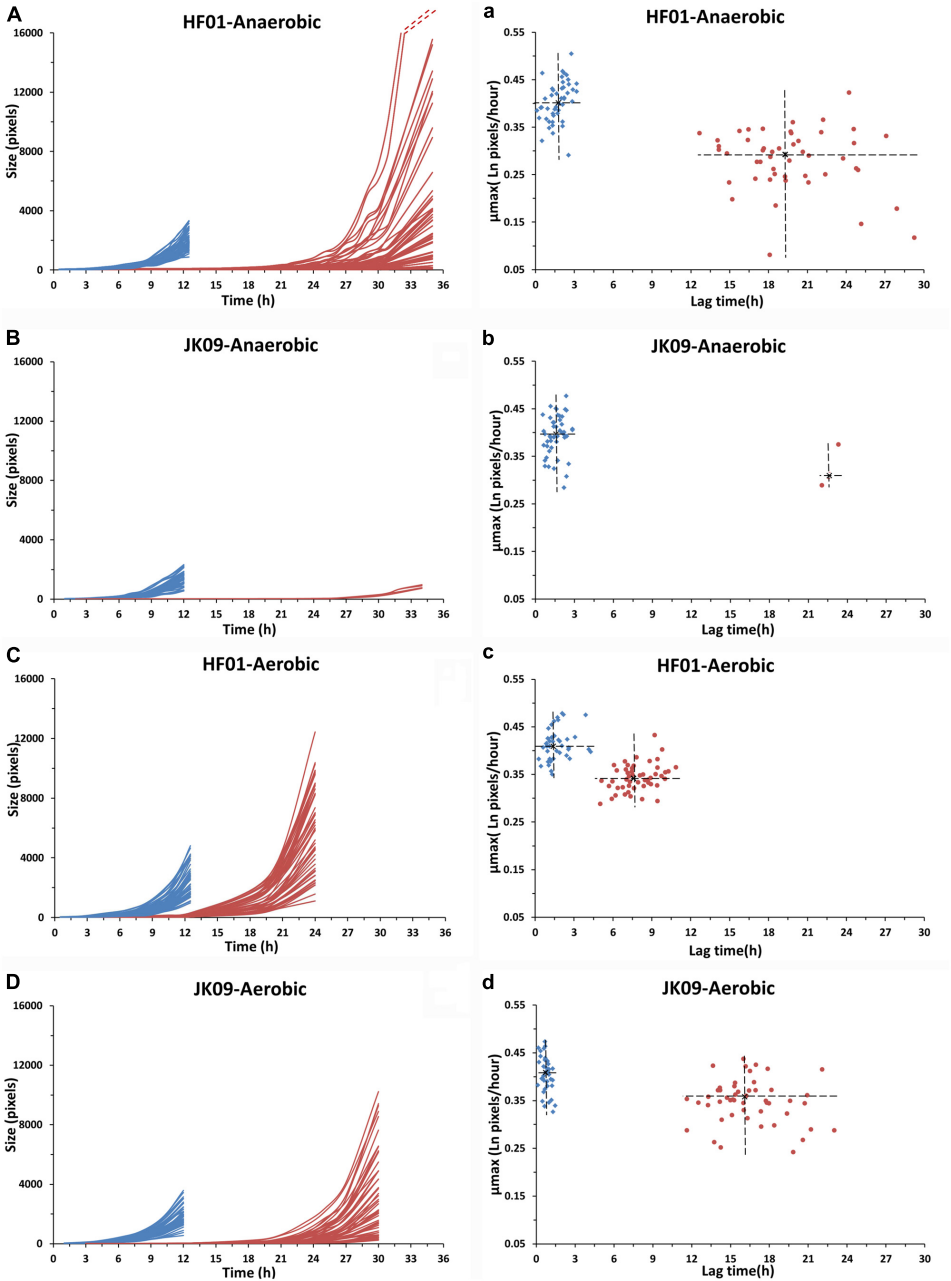
**Growth curves, lag times and μ_**max**_ of individual cells of HF01 and JK09 after different treatments.** Blue: after saline treatment (control); red: after PAA treatment. The dotted red lines indicate that the two fastest growing microcolonies reached approximately 31000 pixels at 35 h. The center of the cross is the median of lag time and μ_max_ **(a–d)**.

The number of individual cells at time zero was from 0 to 14 in each image, and the total individual cell number in 49 images was between 200 and 500. The growth curves of 50 dividing cells for each treatment are shown in **Figures 5A–D** (except for JK09 after anaerobic cultivation after PAA treatment, where only four cells divided). The experiments were repeated, and both strains showed good repeatability (results not shown). The experiment was stopped when the dividing microcolonies merged. It can be seen that for treatment with saline (control, blue lines), the growth of HF01 was similar to that of JK09 after the same cultivation, but with a certain variation in growth of individual cells. After treatment with PAA (red lines), the individual growth curves were quite diverse for both strains.

**FIGURE 6:**
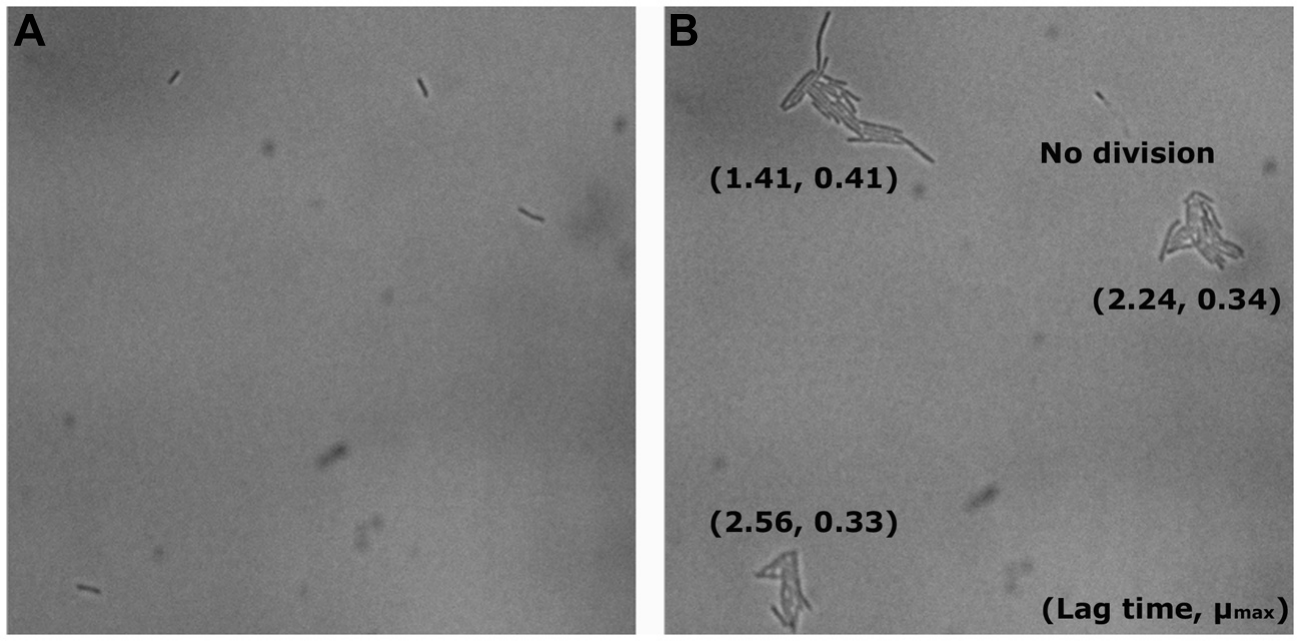
**Example of neighboring cells of JK09 in the control experiment after anaerobic incubation at *T* = 0 **(A)** and *T* = 12 h **(B)****.

The distributions of λ and μ_max_ values, estimated by the Baranyi and Roberts (1994) primary model for 340 microcolonies (14 microcolonies could not fit the model) originating from individual cells, are shown in **Figures 5a–d**. After saline treatments (control, blue dots), the median λ values of JK09 were a little bit smaller than that of HF01 regardless of cultivation. The median λ values for both strains were also slightly smaller after aerobic conditions. The median μ_max_ were almost similar for all control experiments. After PAA treatments (red dots), the median λ values were distinctly bigger and the median μ_max_ were clearly lower compared with the same strain and incubation in the control experiment. For HF01, the aerobic incubation gave smaller median λ values and higher median μ_max_ than the anaerobic incubation. It is impossible to draw conclusions about the influence of incubation on JK09, due to the limited number of dividing cells after anaerobic cultivation. We can also see that both λ and μ_max_ exhibit a significant variability for each experiment, especially after exposure to PAA. However, we could not find a clear correlation between λ and μ_max_. As an example, we show the calculated λ and μ_max_ from three neighbor colonies (**Figure 6**). The cells have almost similar distance to the other two cells, but with a distinct difference in λ and μ_max_.

## DISCUSSION

As previously mentioned, there may be an inverse correlation between hop resistance and oxidation resistance. However, in this study a plasmid cured strain lacking the hop resistance gene horA had a slightly higher sensitivity toward oxidizing compounds compared to the wild type JK09 (**Figure 1**). This does not support the idea of an inverse correlation, but the plasmid cured strain also exhibited similar hop resistance to the wild type (results not shown), which suggests that hor A is neither important for hop resistance nor oxidation resistance.

It was previously found that *Lactobacillus brevis* ATCC 14869 exhibit Smooth(S)-type colonies when grown under anaerobic conditions, whereas the majority of colonies exhibit a Rough(R)-type morphology under aerobic conditions (Jakava-Viljanen et al., 2002). In our study, the two *Lactobacillus brevis* strains HF01 and JK09 had distinctly different morphologies as JK09 exhibited S-type morphology and HF01 exhibited R-type morphology (**Figure 2**). However, their morphologies appeared to be less variable, as they retained the same morphology after PAA treatment, and the morphology was the same after aerobic and anaerobic cultivation. The study of Jakava-Viljanen et al. (2002) indicated that an oxidative environment promotes the formation of R-type colonies, which could suggest that strains with R-type morphology has an increased survival after exposure to oxidative compounds. This is consistent with our findings, where HF01 (R-phenotype) was more tolerant toward PAA.

In addition, our microscopic results suggest that already when microcolonies are formed, a distinct difference in colony morphology can be observed, which may predict the resulting morphology of macrocolonies (**Figures 2A,B and 3**).

It is clear that the microscopic method is more rapid than the CFU method for detection of dividing cells, (Asano et al., 2009). The traditional approach required at least 2 days before visible colonies (consisting of millions of cells) could be detected. In the present study, small colonies continued to appear until five days after the PAA treatment. The origin of these colonies is cells that can be considered ‘hard-to-culture’ (Suzuki, 2011), but our microscopic results suggests that this phenomenon can be attributed to the large variation in lag time of individual cells, after PAA treatment. This could be the reason why some survival rates using the CFU method were 20% lower than that using the microscopic method and with similar big differences between repetitions in the CFU method (**Table 2**). It is possible that some individual cells started division so late that the (macro)colonies were too small to be observed on the last day of the experiment. However, the microscopic method could observe cell elongation and division down to few hours after the beginning of the experiment. The microscopic method also has the potential to provide more details of the growth of individual cells into microcolonies. In our study, we can clearly see the growth dynamics of individual cells (**Figure 3**), where cells divided and eventually formed a microcolony. We also observed in our experiments that a few cells elongated, but then stopped dividing (**Figure 6**). This type of subpopulation cannot be observed with a CFU method, although the cells may possess some amount of biochemical activity. This might be another reason why the survival rates using the CFU method in some cases were 20% lower than the microscopic method.

Several studies have shown that individual cells exhibit heterogeneity in how they deal with stress in the same environment (Lianou et al., 2006; Métris et al., 2008; Muñoz-Cuevas et al., 2013). We observed that there are relatively small differences in the growth curves and lag time of both strains in the control experiments (**Figure 5**), but extensive variation in growth behavior, survival, lag time and maximum growth rate was observed between the two strains after treatment with PAA (**Table 2**, **Figure 5**). This biological variability may be due to the genetic diversity between the strains, but the large variations in the resulting λ and μ_max_ of HF01 and JK09, especially after PAA treatments suggest that there is a phenotypic diversity that cannot be fully explained by presence of genes, as all of the individual cells of a strain can be considered clonal. After exposure to PAA, the lag time of the dividing cells of both strains increased pronouncedly. Interestingly, after the prolonged lag time, some cells of both strains exhibited the same μ_max_ as in the control experiments, whereas other cells grow at a much slower rate. This is interesting, because it suggests that even after repair mechanisms have enabled the individual cells to divide, the resulting daughter cells in a microcolony continue to divide at a rate that is predicted by the initial divisions, even though PAA has been removed. This result is inconsistent with the results from Kutalik et al. (2005), which points out that the extent of the lag phase only influence the first cell cycle and the subsequent division is uncorrelated to the cell history. In addition, we found that the lag time was significantly shorter and the μmax was distinctly higher after the aerobic cultivation for both strains (**Figure 5**). This result indicates that aerobic cultivation makes *Lactobacillus brevis* more tolerant to PAA. It may be because the bacteria have already built up some kind of defense mechanism or repair mechanism in order to protect them against oxygen during the aerobic cultivation. For example, high NADH oxidase activity and NADH peroxidase activity were found in *Lactobacillus brevis* after aerobic cultivation (Sakamoto and Komagata, 1996; Hummel and Riebel, 2003). In addition, there are marked differences in λ and μ_max_ of neighboring microcolonies (**Figure 6**). The differences are not caused by the proximity between the resulting microcolonies, because even after 12 h and several divisions, the resulting microcolonies still have similar distances to the neighboring colonies. It is likely that there would be an interaction between adjacent colonies when they are so close that they must share nutrients (propinquity effect), but it is unlikely that this is the case within the timeframe of our experiments.

The microscopic method used in this study can therefore provide quantitative data suitable for analysis of growth of individual cells. It should be noted that we only quantified the amount of pixels that are covered by microcolonies in two dimensions. When the microcolonies become very big (several hundred cells), the microcolony started to take on the traditional three-dimensional structure (**Figures 4A,C**), where a simple measure of area is no longer adequate. However, during many divisions, the cells are primarily growing on the surface of the agar, as our cells are enclosed between the coverslip that constitutes the bottom of the well and the agar. Additionally, if the microcolonies expand in less coordinated fashion such as HF01, where there can be visible holes inside the microcolony, it is important to subtract the area of the holes to obtain a valid estimate of cell growth.

Propidium iodide is a popular red-fluorescent DNA counterstain for estimating the amount of dead cells in a bacterial population (Bunthof et al., 2001; Rault et al., 2007). Due to its molecular weight and charge, it only penetrates cells with a damaged plasma membrane. As PI does not fluoresce without the presence of DNA, we incorporated PI into our growth matrix, in order to determine when and how, individual cells begin to die. Surprisingly, the large proportion of cells that failed to divide after treatment with PAA, did not exhibit red fluorescence (**Figures 4B,D**). In JK09, the survival after anaerobic cultivation after PAA treatment was around 1%, so we would expect to observe 99% red cells. This observation suggests that PAA does not compromise the membrane integrity of cells that fail to divide, and PI would therefore be a poor indicator of the efficacy of PAA. To rule out potential artifacts in our experiments, we determined that PAA treated cells that were afterward exposed to 70% EtOH (which destroys the membrane) all stained red in our setup (results not shown). As microcolonies started to grow, we observed that a few individual cells turned red, which indicates that these cells have lost their membrane integrity (**Figure 4**). The cells appeared randomly within the microcolony, which does not suggest that they died from nutrition depletion or accumulation of toxic substances. If the cells should die from nutrient depletion or accumulation of toxic substances, it would be expected that the majority of the dead cells would be located in the center of the microcolony, this observation was previously reported by Ryssel et al. (2013).

In conclusion, the investigated beer spoilage LAB after different cultivations exhibit different sensitivity toward PAA, but there is no indication that the tolerance toward PAA is inversely correlated to the potential to spoil beer. On the other hand, the present study demonstrates a novel approach to investigate the formation of microcolonies as an indicator of physiological fitness. The method provides results faster than CFU determination, but seems to correlate very well with CFU. Furthermore, the method provides a tool to investigate the phenotypic heterogeneity of a clonal population, which can be expanded to many interesting aspects.

## Conflict of Interest Statement

The authors declare that the research was conducted in the absence of any commercial or financial relationships that could be construed as a potential conflict of interest.

## Footnotes

1 http://imagej.nih.gov/ij/

2 http://www.combase.cc/index.php/en/
